# PyTSC: A Unified Platform for Multi-Agent Reinforcement Learning in Traffic Signal Control

**DOI:** 10.3390/s25051302

**Published:** 2025-02-20

**Authors:** Rohit Bokade, Xiaoning Jin

**Affiliations:** Department of Mechanical and Industrial Engineering, Northeastern University, Boston, MA 02115, USA; xi.jin@northeastern.edu

**Keywords:** Multi-Agent Reinforcement Learning (MARL), Traffic Signal Control (TSC), intelligent transportation systems (ITSs), urban traffic management

## Abstract

Multi-Agent Reinforcement Learning (MARL) presents a promising approach for addressing the complexity of Traffic Signal Control (TSC) in urban environments. However, existing platforms for MARL-based TSC research face challenges such as slow simulation speeds and convoluted, difficult-to-maintain codebases. To address these limitations, we introduce PyTSC, a robust and flexible simulation environment that facilitates the training and evaluation of MARL algorithms for TSC. PyTSC integrates multiple simulators, such as SUMO and CityFlow, and offers a streamlined API, enabling researchers to explore a broad spectrum of MARL approaches efficiently. PyTSC accelerates experimentation and provides new opportunities for advancing intelligent traffic management systems in real-world applications.

## 1. Introduction

Effective Traffic Signal Control (TSC) is fundamental to urban traffic management, responsible for guiding the movement of vehicles through intersections by controlling traffic lights. The primary goals of TSC are to minimize traffic congestion, enhance traffic flow, and improve safety for both vehicles and pedestrians. Poor TSC optimization leads to increased congestion, fuel consumption, and pollution. Longer wait times at signals lead to increased fuel consumption, which not only exacerbates environmental issues through higher emissions but also results in economic losses due to delays. Moreover, inefficient TSC negatively impacts the quality of life in urban areas, contributing to increased noise and air pollution.

Multi-Agent Reinforcement Learning (MARL) offers a promising approach to tackling these TSC challenges by allowing multiple agents to collaborate within a shared environment [1,2]. In fully cooperative settings, agents work toward a common goal by interacting with their environment and with one another and refine their actions based on the feedback from the environment [3]. MARL’s versatility is demonstrated by its successful application in various domains [4]. For instance, in robotics, MARL has been used to coordinate multiple robots in tasks such as search and rescue operations [5,6]. These successes highlight MARL’s potential to solve complex, multi-faceted problems, making it an ideal candidate for optimizing TSC in dynamic and unpredictable urban environments.

### 1.1. Challenges in Current MARL Research for TSC

The application of MARL to TSC has seen notable advancements [7,8,9,10]. Two simulators, SUMO [11] and CityFlow [12], are widely recognized in this domain, and several open-source tools have been developed to leverage these platforms [13,14,15]. Recent efforts have aimed at merging the TSC environments of both simulators and unifying domain metrics, standardizing evaluation criteria and providing a consistent framework for problem formulation in TSC research [15]. Benchmarks tailored for specific datasets in SUMO have also been developed, supporting a diverse range of MARL algorithms [14]. Despite progress in applying Multi-Agent Reinforcement Learning (MARL) to Traffic Signal Control (TSC), the research community still lacks a unified, modular, and extensible platform that meets the needs of modern MARL algorithms. Most existing TSC simulators are tightly coupled to their frameworks and are not designed to support the flexible integration of advanced MARL methodologies, particularly frameworks like centralized training with decentralized execution (CTDE), which have gained significant traction in recent years.

Existing TSC libraries, such as LibSignal [15] and SUMO-RL [13], offer valuable tools for research but exhibit limitations that can impede advanced MARL studies. LibSignal supports multiple simulators, including SUMO and CityFlow, and provides OpenAI Gym-compatible environments along with baseline methods for TSC tasks. However, it does not natively integrate with state-of-the-art centralized training with decentralized execution (CTDE) frameworks like QMIX or MAPPO, which are crucial for scalable multi-agent coordination. This necessitates additional effort from researchers to adapt LibSignal for such frameworks. Similarly, SUMO-RL focuses exclusively on the SUMO simulator, lacking cross-simulator compatibility. This focus requires researchers to reconstruct their workflows when transitioning to different simulation platforms. Although SUMO-RL offers flexibility in customizing state and reward definitions, its tight coupling with specific MARL algorithms can pose challenges when integrating with alternative learning frameworks. Both libraries adopt architectures that, while functional, may require significant modification to accommodate novel research needs. For instance, adapting LibSignal to support graph-based MARL approaches would involve substantial changes to its core communication modules. Integrating SUMO-RL with CTDE libraries like EPyMARL [16] or MARLLib [17] would similarly demand extensive middleware development. These challenges can divert researchers’ efforts from algorithm innovation to tool adaptation, thereby slowing progress toward effective real-world TSC solutions where decentralized execution and centralized coordination are essential for managing large-scale, dynamic traffic networks.

To address these challenges, we introduce PyTSC—a library specifically designed to overcome the limitations of current TSC platforms. PyTSC provides a clean, modular, and extensible environment that seamlessly integrates with CTDE frameworks, such as EPyMARL and MARLLib. This integration enables researchers to efficiently prototype, train, and evaluate MARL algorithms within a traffic control context. By offering a robust and flexible platform, PyTSC facilitates the exploration of novel TSC solutions, promoting faster experimentation, enhanced reproducibility, and the development of more effective Traffic Signal Control strategies.

### 1.2. Contributions

This work introduces PyTSC, a flexible and efficient simulation environment designed to address these challenges in the MARL-TSC research domain. PyTSC fills a crucial gap in the TSC research ecosystem by providing a platform that simplifies integration, supports cross-simulator comparisons, and offers a clean interface for deploying advanced MARL methods like CTDE. The key contributions of this research are:

1. **Compatibility with Multiple Simulators:** PyTSC supports both SUMO and CityFlow simulators, offering a consistent API that abstracts simulator-specific details. This design facilitates seamless switching between simulators and enables the integration of additional simulators in the future. For example, researchers can train on SUMO and evaluate on CityFlow without modifying their codebase.
2. **Optimized for Speed:** The Retriever module in PyTSC efficiently gathers required information from the simulator after each time step, minimizing simulator queries and improving simulation speeds (https://sumo.dlr.de/docs/FAQ.html#traci (accessed on 18 February 2025)). By batching queries (e.g., vehicle counts, speeds) into a single call, PyTSC significantly reduces overhead compared to standalone simulations, enabling faster experimentation for long-horizon tasks and large-scale networks.
3. **Leveraging Graphical MARL Techniques:** The NetworkParser module processes network files, extracting topological features such as adjacency matrices, lane connectivity graphs, and centrality measures. These features enable graph-based MARL algorithms (e.g., Graph Attention Networks) to model complex traffic networks more effectively. This capability is absent in existing tools like LibSignal and SUMO-RL.
4. **Dataset Aggregation:** PyTSC aggregates commonly use datasets in MARL-TSC, including real-world networks like Hangzhou and Cologne. Additionally, it provides modules like GridGenerator and TripGenerator for creating synthetic networks and traffic demand patterns. These tools allow researchers to test algorithms in controlled environments before deploying them in real-world scenarios.
5. **Unified MARL Formulation for TSC:** While we recognize that we did not develop the Dec-POMDP and Networked MMDP formulations, we advocate for their use in TSC research. These formulations, which allow for decentralized control schemes, are comprehensive and well-suited for deep MARL techniques in TSC. By promoting these formulations, we aim to standardize nomenclature and problem formulations in the TSC field, facilitating clearer communication and collaboration among researchers.
6. **Experiments with CTDE MARL Frameworks:** PyTSC integrates seamlessly with state-of-the-art CTDE frameworks like EPyMARL and MARLLib. As a demonstration, we present experiments with several MARL techniques, including QMIX, VDN, and MAA2C, which follow the CTDE paradigm. These experiments highlight PyTSC’s ability to support advanced MARL algorithms in complex traffic networks.
7. **Modular and Extensible Design:** PyTSC’s modular architecture allows researchers to easily customize observation spaces, reward functions, and action spaces by extending abstract classes like BaseObservationSpace and BaseRewardFunction. This flexibility enables rapid prototyping and experimentation with novel MARL algorithms without modifying the core codebase.

In summary, PyTSC is not merely a tool but a significant advancement in TSC research. By integrating MARL into a well-structured environment, PyTSC has the potential to redefine TSC research, propelling both academic studies and practical applications.

## 2. The PyTSC Framework

**Figure 1:**
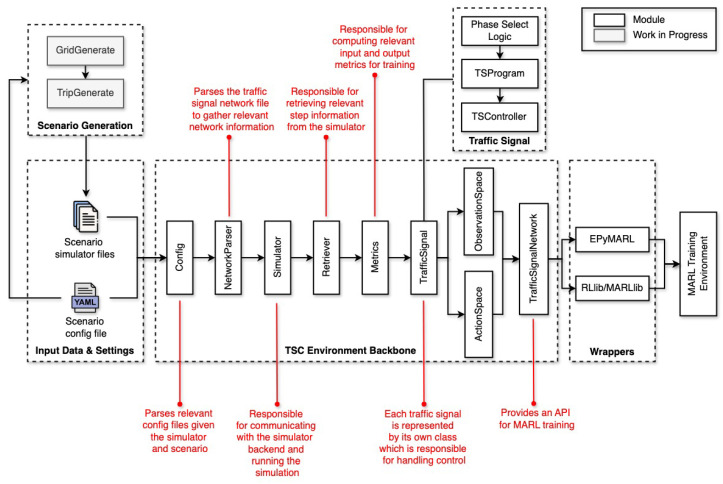
Overview of PyTSC architecture.

PyTSC’s framework (Figure 1) is designed to bridge the gap between traffic simulations and MARL. The library is available at https://github.com/rbokade/pytsc (accessed on 18 February 2025). The library, available at https://github.com/rbokade/pytsc (accessed on 18 February 2025), simplifies the process of developing and testing MARL algorithms for traffic signal control.

The framework’s key features include the following:

- **Support for Diverse Simulator Backends:** PyTSC integrates a consistent API for two renowned simulator backends: SUMO [11] and CityFlow [12]. Simulator specific classes like ConfigParser, Retriever, Simulator, and TrafficSignal allow for processing the input from the simulators into a common format which can then be used in the TrafficSignalNetwork environment class. This uniform interface not only maintains consistency across simulators but also offers a foundation for researchers to incorporate additional simulator backends as needed.
- **Customizable:** PyTSC’s modular design lets researchers adapt it to their needs. Users can experiment with various traffic signal network settings by choosing from existing modules or extend them to suit their own needs. For example, TLSFreePhaseSelectLogic and TLSRoundRobinPhaseSelectLogic allow users to select either adaptive phase selection or fix it to a round robin strategy. Users can also modify the information required by the MARL algorithm by simply extending BaseObservationSpace, BaseActionSpace, and BaseRewardFunction according to their needs.
- **Optimized Performance:** The framework is optimized to gather essential metrics from the backend simulator in a single query after each simulation step. This streamlined approach minimizes redundant queries, leading to faster simulation processes. For SUMO simulator backends, it uses subscriptions https://sumo.dlr.de/docs/FAQ.html#traci (accessed on 18 February 2025) to further speed up simulations.
- **Integration of Static Network Features:** The NetworkParser module automatically extracts structural details from traffic network files of the chosen simulator. Key outputs include (1) intersection metadata: IDs, positions, and lane configurations for all traffic signals; (2) adjacency matrices: a graph representation of intersections, showing which signals are directly connected; and (3) lane connectivity: maps how lanes link between intersections, critical for modeling vehicle movement, etc. These features enable MARL algorithms to leverage the traffic network’s topology. For example, graph neural networks (GNNs) can use adjacency matrices to model coordination between signals, while centrality metrics help prioritize control at critical junctions. This automation eliminates manual network analysis, streamlining research workflows.
- **Compatibility with MARL Training Frameworks:** PyTSC works with popular MARL libraries like rllib [18], PyMARL [19], and EPyMARL [16], making it easy to apply advanced algorithms such as QMIX or MAPPO. Researchers can focus on developing new methods without rebuilding simulation environments from scratch.

## 3. Experiments

### 3.1. Traffic Signal Network Scenarios

We have curated 10 open-source scenarios most commonly used by researchers while applying MARL techniques to TSC. These scenarios, widely adopted by researchers in the field, encompass both synthetic and real-world traffic networks and are compatible with both CityFlow and SUMO simulators. A detailed overview of these scenarios is presented in Table 1 (https://github.com/cts198859/deeprl_network (accessed on 18 February 2025), https://github.com/LucasAlegre/sumo-rl (accessed on 18 February 2025), https://github.com/traffic-signal-control/sample-code/ (accessed on 18 February 2025), https://github.com/Pi-Star-Lab/RESCO/ (accessed on 18 February 2025)).

**Figure 2:**
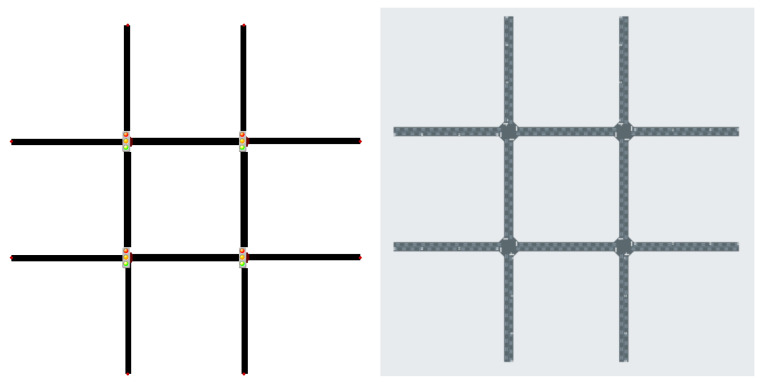
2×2 Grid SUMO (**left**) CityFlow (**right**).

**Figure 3:**
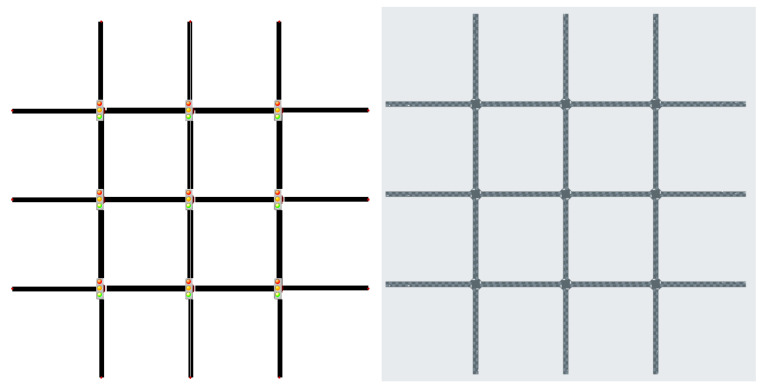
3×3 Grid SUMO (**left**) CityFlow (**right**).

**Figure 4:**
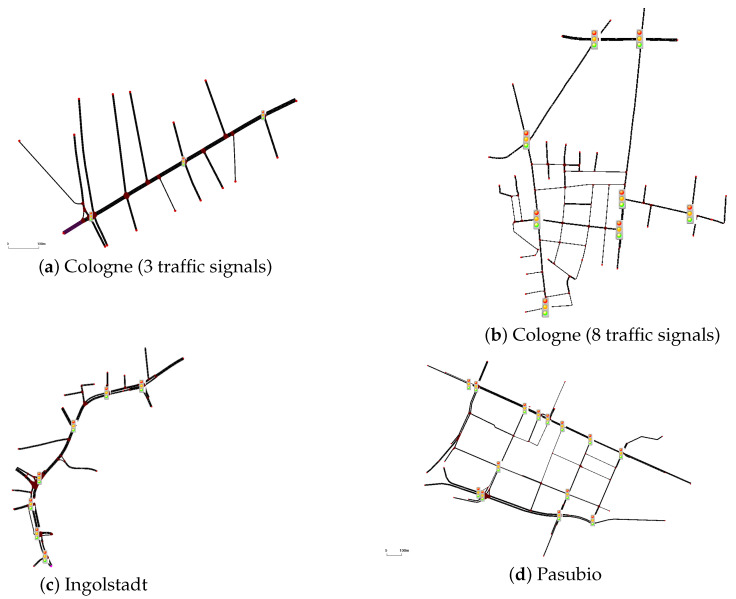
Real-world environments for SUMO.

**Figure 5:**
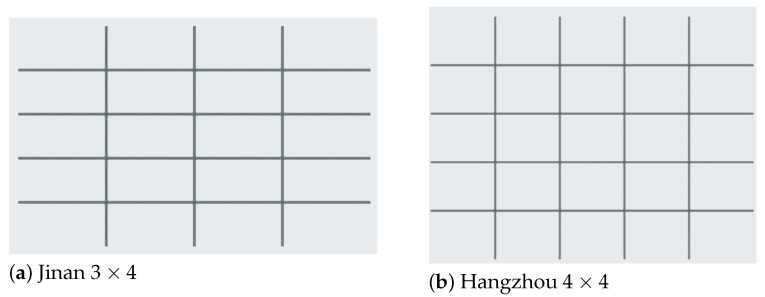
Real-world environments for CityFlow.

The scenarios in Table 1 offer a mix of synthetic grid networks and real-world traffic settings. Synthetic grids, from 2×2 (Figure 2) and 3×3 (Figure 3), provide a controlled environment with homogeneous agents for testing MARL techniques. In contrast, real-world scenarios from cities like Cologne, Ingolstadt, and Monaco present urban complexities with heterogeneous agents, ranging from 3 to 16 (Figure 4 and Figure 5). PyTSC includes large-scale networks like Hangzhou (16 signals) and Cologne 8, which test MARL algorithms in dense urban environments. These scenarios feature heterogeneous traffic flows and dynamic routing (in SUMO), reflecting real-world complexity. For example, Hangzhou’s grid network challenges algorithms to optimize coordination across 16 intersections, while Cologne 8 tests adaptability in irregular, high-traffic networks. Additionally, PyTSC’s compatibility with both SUMO and CityFlow allows researchers to benchmark algorithms across multiple simulators, ensuring robust evaluation. SUMO’s dynamic routing allows vehicles to adapt to congestion, enabling global optimization in networks like Cologne. In contrast, CityFlow’s static routes simplify coordination, making it suitable for controlled experiments. PyTSC’s compatibility with both simulators ensures flexibility in evaluating algorithms under different traffic dynamics.

These scenarios form a robust benchmarking platform for MARL in TSC, covering diverse network types and complexities. By including real-world networks like Hangzhou and Cologne, PyTSC enables researchers to develop and test MARL algorithms that can be directly applied to real-world traffic management systems, improving urban mobility and reducing environmental impact.

### 3.2. Traffic Signals as Agents

In MARL, under fully-cooperative settings, agents learn to achieve a common goal or maximize their individual rewards through interaction with the environment and each other. In the context of TSC, MARL provides a framework for developing Traffic Signal Control strategies that can adapt to changing traffic patterns and optimize flow. Traffic signals are modeled as agents whose goal is to choose control the traffic lights to minimize congestion throughout the traffic signal network.

#### 3.2.1. Decentralized Partially Observable Markov Decision Processes (Dec-POMDPs)

Dec-POMDPs model multi-agent systems [20] where agents interact in a decentralized way under limited visibility. This is pertinent for analyzing traffic signal control.

**Definition.**  A Dec-POMDP is defined as a tuple ⟨*N*, *S*, {*Ai*}, {*Oi*}, *T*, {Ω*i*}, *R*⟩, where:

- *N*: A finite set of *n* agents, N≡{1,…,n};
- *S*: A finite set of states that describe the global state of the environment;
- $\left\{A_{i}\right\}$: A finite set of actions for each agent *i*, with the joint action space $A\equiv \{A_{1}\times \ldots \times A_{n}\}$;
- $\left\{O_{i}\right\}$: A finite set of observations for each agent *i*, with the joint observation space $O\equiv \{O_{1}\times \ldots \times O_{n}\}$;
- T:S×A↦Δ(S): A state transition function that maps the current state and joint action to a probability distribution over the next states;
- $\left\{\Omega_{i}:S\times A\mapsto \Delta \left(O_{i}\right)\right\}$: An observation function for each agent, mapping the current state and joint action to a probability distribution over individual observations;
- R:S×A↦R: A reward function that maps a global state and joint action to a real-valued reward.

By capturing the decentralized nature and partial observability inherent in urban traffic systems, Dec-POMDPs offer a foundation for designing MARL frameworks that can lead to more responsive and efficient traffic signal control. We adopt Dec-POMDPs because they are the foundation for most state-of-the-art MARL algorithms, such as QMIX and MAPPO, ensuring compatibility with existing research. Additionally, Dec-POMDPs naturally support decentralized execution, which is essential for scaling to large urban networks. While PyTSC primarily focuses on Dec-POMDPs, its support for graph-based communication (e.g., via the NetworkParser) allows researchers to implement networked MDP algorithms if desired. This flexibility ensures PyTSC can accommodate a wide range of MARL approaches.

#### 3.2.2. Observation Space

Each traffic signal has a field of view up to 50 m, within which it can acquire traffic flow information. This range corresponds to the sensory data typically obtained from common real-world traffic sensors. The observation for each traffic signal comprises the following components: the number of vehicles ${\left\{n_{l}\right\}}_{l=1}^{L_{i}}$, the average normalized speed of the vehicles ${\left\{s_{l}\right\}}_{l=1}^{L_{i}}$, the number of halted vehicles (queue lengths) ${\left\{q_{l}\right\}}_{l=1}^{L_{i}}$, and the current phaseID of the traffic signal. Here, $L_{i}\in L$ represents the set of incoming lanes for traffic signal *i*, while *L* denotes the set of all lanes in the network.

#### 3.2.3. Action Space

For each traffic signal, *i* determines its action $a_{i}$ by selecting a green phase from a predefined set of operational phases. The system permits a traffic signal to either maintain its current green phase or transition to a new one, after which a mandatory yellow phase is systematically triggered by the simulation environment. The timing for phase transitions and the duration of yellow phases are uniformly fixed at 5 s intervals throughout the simulation.

#### 3.2.4. Reward Function

In Traffic Signal Control scenarios, various metrics can be utilized to define rewards. In this study, we adopt queue length $q_{l}$ as the performance metric for the traffic signal controller. This choice is motivated by its simplicity and its ability to provide an instantaneous feedback signal. The objective is to minimize the number of stopped vehicles across the network, which is expressed as follows:

$$r_{t}=\sum_{l\in L}q_{l}$$

where $r_{t}\in R$ is the global reward and l∈L represents the lanes in the network.

### 3.3. MARL Frameworks

For benchmarking CTDE algorithms in TSC, we employ EPymarl, an extension of the widely recognized Pymarl library. EPymarl encompasses a broad spectrum of algorithms under the reinforcement learning paradigms of Q-learning, actor-critic, and policy gradient methods.

A key consideration in our framework design is the selection of the Dec-POMDP formulation over networked MDPs. Dec-POMDP better models real-world Traffic Signal Control scenarios where each agent (traffic light) has limited observability due to occlusions and sensor noise. Unlike networked MDPs, which assume global state knowledge and structured inter-agent communication, Dec-POMDP allows agents to learn implicit coordination strategies based only on local observations, making it more suitable for real-world deployment where centralized communication may be unreliable or infeasible (https://github.com/uoe-agents/epymarl/ (accessed on 18 February 2025), https://github.com/oxwhirl/pymarl (accessed on 18 February 2025)).

**Figure 6:**
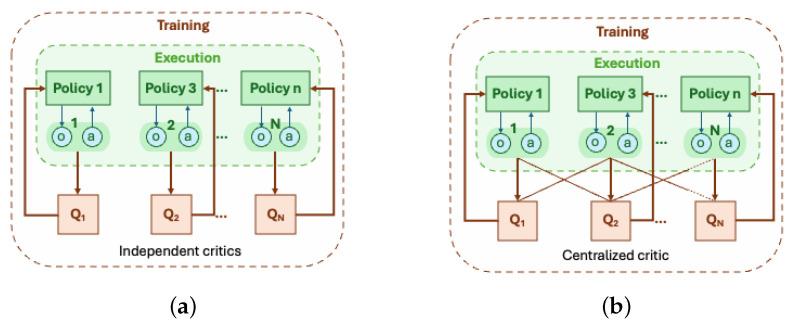
Comparison of independent and centralized critic approaches in MARL training. (**a**) Independent critics: Each agent maintains its own critic (e.g., IQL and IA2C) and updates it based on local observations and rewards, leading to decentralized learning without explicit coordination. (**b**) Centralized critic: A shared critic evaluates global action values (e.g., QMIX and MAA2C) using information from all agents, allowing for centralized training while maintaining decentralized execution.

IQL is a decentralized, value-based method that operates off-policy. VDN and QMIX are both value-based but differ in centralized training, with QMIX using a mixing network to combine local Q-values, making it particularly effective for irregular network topologies with varying intersection connectivity. IA2C and MAA2C, both actor-critic methods, integrate value-based and policy-based learning. IA2C operates without centralized value estimation (see Figure 6a), whereas MAA2C leverages centralized value estimation (see Figure 6b) while maintaining decentralized execution. This makes MAA2C more adaptable to dynamic traffic conditions, such as mixed urban and arterial road networks, where static methods struggle to generalize.

Our selection focuses on the most prevalent MARL frameworks, as detailed in Table 2. Overall, by integrating a variety of MARL techniques, PyTSC provides a flexible and scalable platform for studying adaptive traffic signal control. By benchmarking these algorithms, we aim to provide insights into their applicability, strengths, and limitations within the context of TSC.

### 3.4. Evaluation Protocols

The performance of the Multi-Agent Reinforcement Learning (MARL) algorithms was evaluated through a carefully designed training and testing process. Each episode during training was constrained to 360 simulation seconds, which corresponds to 72 time steps. This time frame was selected to ensure a balance between simulation length and computational efficiency. Therefore, one simulation hour consisted of 10 episodes, which allowed the MARL algorithms to interact with the environment multiple times within a relatively short period (see Table 3 for a detailed breakdown of time steps and simulation periods).

In total, the algorithms were trained for 4.32 million time steps, which corresponds to 36,000 episodes. This extensive training schedule, equivalent to 6000 h of simulated traffic, provided ample opportunity for the agents to learn optimal strategies for traffic signal control. To assess the generalization capabilities of the trained models, evaluation was performed every 200 episodes. For each evaluation cycle, the models were tested over 10 episodes to ensure that performance was not merely due to overfitting but could be replicated under various conditions (see Table 3 for details on testing intervals and length).

To optimize the MARL algorithms, hyperparameter tuning was conducted using EPyMARL’s grid search feature on smaller synthetic grid networks, such as 2 × 2 and 3 × 3 grids. This approach allowed for systematic exploration of hyperparameter combinations, ensuring that the selected configurations promoted learning stability and convergence before scaling to larger networks. Key hyperparameters, including buffer size, learning rate, entropy coefficient, and target update intervals, were tuned based on their impact on training efficiency and robustness. The grid search method was chosen to balance exploration and exploitation, automating the selection of optimal values while minimizing manual trial-and-error. Details of the chosen hyperparameters can be found in Table 4.

All experiments were conducted on Northeastern University’s High-Performance Computing (HPC) Discovery cluster. Each experiment utilized 8 single-core CPUs, with four parallel environments running simultaneously to gather data efficiently. This setup allowed for significant computational power and parallelization, enabling the MARL algorithms to process multiple simulations concurrently and reduce the overall time required for training and evaluation.

## 4. Results

The performance of various MARL algorithms was evaluated across different environments using SUMO and CityFlow simulators. These environments ranged from simple synthetic grid networks to more complex real-world networks, such as Jinan, Hangzhou, Pasubio, and Cologne. The results highlight several key factors that affect algorithmic performance, including network topology and the simulation platform.

### 4.1. Performance on Synthetic Grid Networks

#### 4.1.1. SUMO Grids

**Figure 7:**
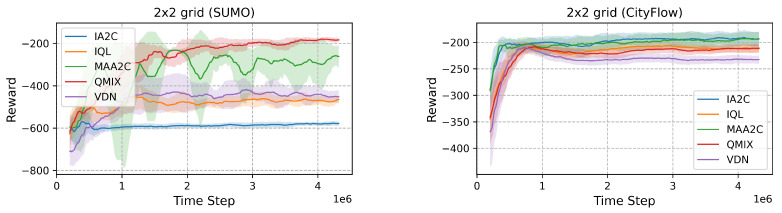
Comparison of rewards in different environments.

Centralized methods, such as QMIX and MAA2C, show superior performance in synthetic SUMO grids compared to decentralized approaches like IQL and IA2C (see Figure 7. Specifically, QMIX achieves the lowest queue length in the 2 × 2 grid (276.69) and the shortest travel time (188.78), significantly outperforming decentralized methods such as IQL, which has a queue length of 486.47 and a travel time of 280.34. In the 3 × 3 grid, QMIX maintains its advantage with a queue length of 361.57 and a delay of 0.534, outperforming IA2C, which has a queue length of 411.47 and a delay of 0.556. These results highlight that centralized algorithms excel in optimizing traffic flows in environments with dynamic routing.

**Figure 8:**
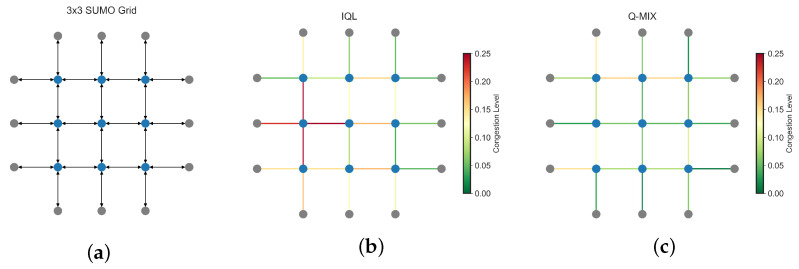
Comparison of MARL approaches in a 3×3 SUMO grid. Congestion is measured as the average lane occupancy over the period of 1 h, with red indicating high congestion and green representing smooth traffic flow. (**a**) Network representation (**b**) Congestion Map (IQL) (**c**) Congestion Map (Q-MIX).

Figure 8 visualizes congestion levels in 3 × 3 SUMO grid. The network structure (Figure 8a) is extracted by PyTSC’s NetworkParser module, where nodes represent intersections and edges denote traffic flow. Figure 8b,c depict average lane occupancy for IQL and QMIX, respectively. Congestion, measured as lane occupancy over time, is color-coded, with red indicating heavy congestion and green representing smooth traffic. In IQL (Figure 8b), key intersections experience severe congestion due to a lack of coordination among agents. In contrast, QMIX (Figure 8c) significantly reduces congestion, leveraging a centralized critic for better traffic optimization. These visual trends align with numerical results, where QMIX achieves lower queue lengths and travel times.

#### 4.1.2. CityFlow Grids

In CityFlow, the performance gap between centralized and decentralized methods is narrower due to the simpler traffic simulation. In the 2 × 2 grid, QMIX achieves a queue length of 224.97, comparable to IQL, which achieves 222.72, with travel times of 227.97 and 223.13, respectively. Similarly, in the 3 × 3 grid, queue lengths for QMIX and IQL are closer, with QMIX achieving 484.57 and IQL achieving 445.10. Delays and travel times also show marginal differences between these methods. These results suggest that CityFlow reduces the coordination requirements, leveling the performance across algorithms.

### 4.2. Performance on Real-World Traffic Networks

#### 4.2.1. Jinan and Hangzhou

**Figure 9:**
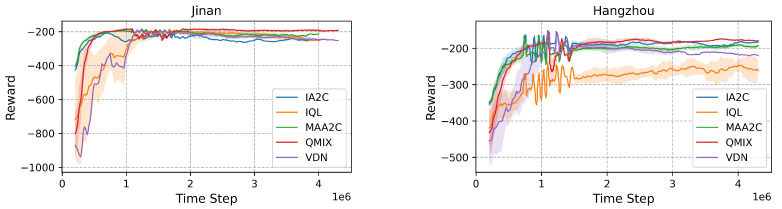
Comparison of rewards in Jinan and Hangzhou environments.

**Figure 10:**
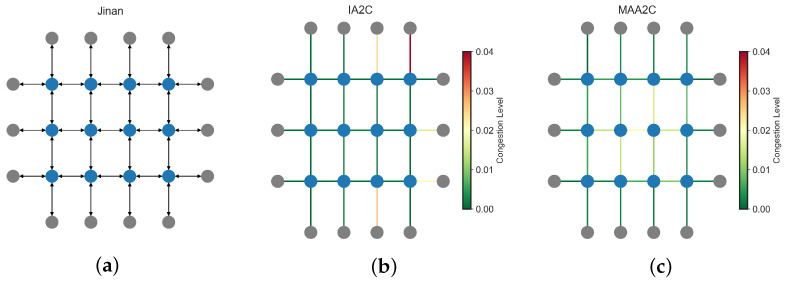
Comparison of MARL performance in Jinan. Congestion is measured as the average lane occupancy, with red indicating higher congestion. (**a**) Network representation (**b**) Congestion Map (IA2C) (**c**) Congestion Map (MAA2C).

Centralized methods demonstrate clear advantages in dense urban networks with high traffic complexity (see Figure 9). Figure 10 provides congestion maps for Jinan, where IA2C (Figure 10b) exhibits more congested corridors compared to MAA2C (Figure 10c). This aligns with numerical results showing that QMIX achieves the lowest queue length (231.56) and travel time (308.67), outperforming IA2C (queue length: 248.49, travel time: 311.91). In Hangzhou, QMIX also achieves lower delays (0.554) compared to IQL (0.585), reinforcing the advantage of centralized coordination in managing dense urban traffic effectively.

#### 4.2.2. Cologne

**Figure 11:**
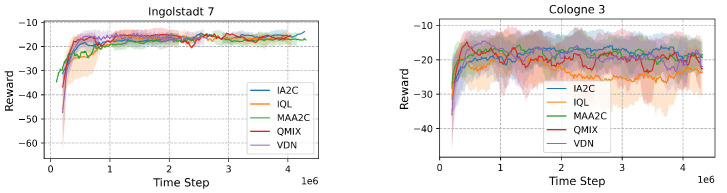
Comparison of rewards in Ingolstadt and Cologne environments.

The performance of algorithms in Cologne depends heavily on the network topology. In the simpler three-signal network, decentralized IA2C achieves a queue length of 18.67, which is comparable to centralized QMIX’s 20.49. In the more complex eight-signal network, centralized QMIX achieves a significant advantage with a queue length of 17.35, compared to IQL, which achieves 19.95 (see Figure 11). These findings highlight that while decentralized methods are effective in simple networks, centralized approaches are critical for managing larger, more complex grids.

#### 4.2.3. Ingolstadt and Pasubio

In irregular and sprawling networks, centralized methods outperform decentralized ones due to better coordination capabilities. In Ingolstadt, QMIX achieves a delay of 0.173, outperforming IQL, which achieves 0.184 (see Figure 11). In Pasubio, QMIX achieves the lowest queue length of 297.79, compared to IA2C’s 340.11. Centralized algorithms effectively handle bottlenecks and uneven traffic distribution in such networks.

### 4.3. Insights from Performance Metrics

Table 5, Table 6 and Table 7 summarize key performance metrics, such as queue lengths, delays, and travel times, across the evaluated networks. The results reveal several key trends:

- **Queue Length:** Centralized algorithms generally achieve shorter queue lengths, particularly in more complex networks like Pasubio and Cologne 8, where coordination across intersections is critical for maintaining traffic flow.
- **Delays:** Across all scenarios, centralized approaches reduce delays more effectively than decentralized ones. The difference is especially notable in SUMO environments, where dynamic routing allows centralized algorithms to adapt to real-time traffic conditions more effectively.
- **Travel Time:** In networks with higher complexity, such as Jinan and Hangzhou, centralized approaches minimize travel time more effectively than decentralized methods. However, in simpler networks like CityFlow’s 2 × 2 grid, the travel times across all algorithms are comparable, suggesting that centralized coordination offers diminishing returns in less complex environments.

**Table 5:** Mean and standard error of metrics for various controllers across scenarios.

**Metric**	**MARL Controllers**					**Rule-Based Controllers**			
	**IQL**	**IA2C**	**VDN**	**QMIX**	**MAA2C**	**Fixed**	**Greedy**	**Max Pres.**	**SOTL**
2×2 SUMO									
Queue	486.47	588.11	475.32	276.69	333.48	464.87	556.88	556.88	556.88
Delay	0.583	0.586	0.586	0.579	0.564	0.59	0.67	0.67	0.67
Travel Time	280.34	329.45	274.92	188.78	239.00	325.02	261.45	261.45	261.45
3×3 SUMO									
Queue	500.98	411.47	520.54	361.57	449.05	560.08	656.85	656.85	656.85
Delay	0.585	0.556	0.588	0.534	0.554	0.61	0.62	0.62	0.62
Travel Time	140.31	139.92	137.19	146.76	143.27	180.52	166.68	166.68	166.68
2×2 CityFlow									
Queue	222.72	201.71	239.34	224.97	205.05	314.08	281.75	261.41	377.43
Delay	0.606	0.600	0.611	0.605	0.598	0.6603	0.6466	0.6362	0.6993
Travel Time	223.13	191.88	239.19	227.97	209.88	325.02	219.48	203.31	437.33
3×3 CityFlow									
Queue	445.10	396.93	520.17	484.57	404.01	621.36	557.96	458.11	738.95
Delay	0.65	0.63	0.67	0.68	0.64	0.69	0.66	0.64	0.74
Travel Time	285.17	249.42	312.03	290.67	288.64	389.21	277.8	246.52	498.33

**Table 6:** Mean and standard error of metrics for various controllers across scenarios.

**Metric**	**MARL Controllers**					**Rule-Based Controllers**			
	**IQL**	**IA2C**	**VDN**	**QMIX**	**MAA2C**	**Fixed**	**Greedy**	**Max Pres.**	**SOTL**
Jinan									
Queue	278.55	248.49	269.14	231.56	221.79	413.55	210.17	228.2	663.39
Delay	0.479	0.467	0.476	0.469	0.470	0.54	0.47	0.49	0.53
Travel Time	320.51	311.91	323.85	308.67	307.00	354.96	287.33	294.97	425.01
Hangzhou									
Queue	286.11	203.24	234.48	208.95	211.18	301.93	171.16	177.93	354.57
Delay	0.585	0.548	0.565	0.554	0.559	0.63	0.56	0.58	0.61
Travel Time	344.72	315.13	327.71	319.03	319.11	356.65	292.77	296.73	364.87

**Table 7:** Mean and standard error of metrics for various controllers across scenarios.

**Metric**	**MARL Controllers**					**Rule-Based Controllers**			
	**IQL**	**IA2C**	**VDN**	**QMIX**	**MAA2C**	**Fixed**	**Greedy**	**Max Pres.**	**SOTL**
Cologne 3									
Queue	23.46	18.67	19.55	20.49	18.80	51.52	53.29	53.29	53.29
Delay	0.334	0.300	0.316	0.319	0.301	0.5	0.54	0.54	0.54
Travel Time	227.11	227.23	230.06	227.46	230.87	220.7	210.54	210.54	210.54
Cologne 8									
Queue	19.95	18.32	20.77	17.35	17.39	41.71	73.87	73.87	73.87
Delay	0.190	0.185	0.184	0.171	0.183	0.26	0.3	0.3	0.3
Travel Time	356.59	356.23	356.08	356.96	356.52	358.14	339.59	339.59	339.59
Ingolstadt 7									
Queue	18.45	17.46	20.35	17.38	19.19	107.06	51.8	50.56	44.16
Delay	0.184	0.169	0.190	0.173	0.177	0.42	0.37	0.36	0.33
Travel Time	192.82	193.35	193.04	193.03	192.74	174.66	183.37	184.47	186.09
Pasubio									
Queue	335.29	340.11	307.60	297.79	322.94	304.82	330.26	330.26	330.26
Delay	0.35	0.33	0.30	0.29	0.30	0.34	0.39	0.39	0.39
Travel Time	303.67	310.88	322.29	321.03	327.31	326.0	292.09	292.09	292.09

### 4.4. Topology-Dependent Performance

The topology of the traffic network plays a crucial role in determining the success of different MARL algorithms. In simpler, grid-like networks, such as the three-signal Cologne setup, decentralized algorithms can perform on par with centralized methods. However, as network complexity increases, the advantages of centralized coordination become more pronounced. In networks like Pasubio and Ingolstadt, which feature irregular layouts and complex traffic flows, centralized approaches like QMIX and MAA2C outperform decentralized methods by a significant margin. This suggests that the choice of algorithm should be informed by the specific characteristics of the traffic network. Centralized algorithms are more suitable for complex, irregular networks with high traffic density, while decentralized approaches may suffice in simpler, more uniform networks.

## 5. Conclusions

In this work, we introduced PyTSC, a versatile and extensible library designed to address the significant gaps in MARL-based TSC research. By enabling compatibility with multiple simulators, such as SUMO and CityFlow, PyTSC provides a unified API that simplifies the development, testing, and benchmarking of MARL algorithms for TSC. Additionally, its optimized architecture facilitates faster simulation speeds, allowing researchers to focus on algorithmic innovations rather than technical integration.

PyTSC’s integration with modern CTDE frameworks, such as EPyMARL and MARLLib, allows researchers to prototype and evaluate advanced MARL techniques within a traffic control setting. This fills a critical gap in the existing research ecosystem, where tools are either too domain-specific or lack the flexibility required for seamless experimentation. With its modular and extensible design, PyTSC establishes a foundation for more consistent, reproducible, and scalable MARL research in the realm of traffic signal control. This contributes directly to advancing smarter, more adaptive traffic management solutions. The performance evaluations across both synthetic and real-world traffic networks demonstrate the library’s effectiveness and versatility, underscoring its potential to drive innovation in TSC systems.

Beyond technical advancements, PyTSC contributes to broader urban mobility and sustainability goals. By optimizing traffic signal coordination, PyTSC helps reduce congestion, lower vehicle delays, and improve overall traffic flow, leading to fuel savings and reduced emissions [21]. This, in turn, enhances air quality, decreases noise pollution, and improves daily commute experiences for drivers and pedestrians [22]. The ability to effectively manage real-world traffic using MARL-based approaches can lead to smarter cities with improved infrastructure utilization, supporting future advancements in intelligent transportation systems (ITSs) and autonomous vehicle coordination [23].

## 6. Future Work

Looking ahead, PyTSC opens several avenues for future development and exploration:

- **Incorporation of Additional MARL Algorithms:** Future iterations of PyTSC will include policy-based MARL algorithms such as MADDPG, IPPO, and MAPPO, expanding its capabilities for more complex traffic control strategies.
- **Diverse Traffic Flow Generation:** We aim to introduce more diverse traffic flow generation models, including both synthetic and real-world flow patterns, to better simulate the variety of traffic scenarios seen in urban environments.
- **Synthetic Network Generation Modules:** Expanding the tools for generating synthetic traffic signal networks will enable researchers to test MARL algorithms in even more controlled and complex environments, beyond the existing benchmarks.
- **Tailored Neural Network Architectures:** While the current version of PyTSC relies on out-of-the-box neural architectures, future updates will explore the integration of specialized models, such as Graph Neural Networks (GNNs), that can leverage the graphical structure of traffic networks to optimize decision making.
- **Simulating Sensor Noise and Real-World Constraints:** To bridge the gap between simulation and real-world deployment, future versions of PyTSC will incorporate sensor noise models to simulate noisy observations and real-world uncertainties. This will help evaluate MARL algorithms in more realistic urban settings, accounting for imperfect data from traffic cameras, loop detectors, or GPS-based sensors.
- **Extending Simulator Compatibility:** We also plan to integrate additional traffic simulators into the PyTSC framework, ensuring that the platform remains adaptable to emerging technologies and research needs in the field of intelligent transportation systems.

By continuously evolving PyTSC, we aim to provide a robust and dynamic platform that will foster innovation in MARL-based Traffic Signal Control and further enhance the real-world applicability of these technologies.

## Figures and Tables

**Table 1:** Scenarios included in PyTSC.

Simulator	Scenario	Network Type	Agent Type	Total Agents
SUMO	2×2 grid	Synthetic	Homogeneous	4
	3×3 grid	Synthetic	Homogeneous	9
	Cologne 3	Real-world	Heterogeneous	3
	Ingolstadt 7	Real-world	Heterogeneous	7
	Cologne 8	Real-world	Heterogeneous	8
	Pasubio	Real-world	Heterogeneous	8
CityFlow	2×2 grid	Synthetic	Homogeneous	4
	3×3 grid	Synthetic	Homogeneous	9
	Jinan (3×4 grid)	Real-world	Homogeneous	12
	Hangzhou (4×4 grid)	Real-world	Homogeneous	16

**Table 2:** MARL algorithms for benchmarking.

Algorithm	Centralized Training	Off-/On-Policy	Value-Based	Policy-Based
IQL	✗	✗	✓	✗
IA2C	✗	✓	✓	✓
VDN	✓	✗	✓	✗
QMIX	✓	✗	✓	✗
MAA2C	✓	✓	✓	✓

Note: ✓ indicates that the property is present in the algorithm, while ✗ indicates that the property is absent.

**Table 3:** Breakdown of hyperparameters used for benchmarking.

Metric	Time Step	Simulation Seconds	Simulation Hours
Step	1	5	0.083
Episode limit	72	360	0.10
Training (length)	4.32 M	21.6 M	6000
Test (interval)	14,400	7200	2
Test (length)	720	3600	1

**Table 4:** Hyperparameters: grid search and selected values.

Hyperparameter	Grid Search Values	Selected Value
Buffer size (episodes)	{500, 1000, 5000}	5000
Hidden dimension	{64, 128}	64
Learning rate	{0.001, 0.0005, 0.0001}	0.0005
Epsilon anneal (steps)	{50,000, 100,000, 200,000}	50,000/100,000
Target update (episodes)	{100, 200, 500}	200
Entropy coeff.	{0.01, 0.05, 0.1}	0.01

## Data Availability

The data presented in this study are available as an open-source github repository at https://github.com/rbokade/pytsc (accessed on 18 February 2025).
